# Neural and autonomic regulation during brief mindfulness and relaxation interventions in clinical populations: a multimodal MEG study protocol

**DOI:** 10.3389/fnins.2026.1804069

**Published:** 2026-06-11

**Authors:** Sabrina Diny, Fabian Schmidt, Andreas Wutz, Nathan Weisz, Thomas Probst

**Affiliations:** Department of Psychology, University of Salzburg, Salzburg, Austria

**Keywords:** ADHD, brain–body coupling, depression, magnetoencephalography, mindfulness, mental health interventions, relaxation

## Abstract

Mental disorders pose a major and growing challenge for health care systems worldwide, marked by persistent treatment gaps and limited access to psychotherapeutic care. Mind–body interventions such as mindfulness and relaxation practices are widely used in clinical contexts as low-threshold strategies to support stress regulation and psychological well-being. Despite their broad application, the mechanisms underlying their acute effects remain insufficiently understood, particularly regarding brain–body interactions. This study protocol describes a prospective multimodal investigation of regulation during brief mindfulness-and relaxation-based interventions in clinical populations (at least *n* = 15 adults with depression according to SCID-5-CV and at least *n* = 15 adults with adult ADHD according to SCID-5-CV). Using a standardized within-subject experimental paradigm, magnetoencephalography (MEG) will be combined with electrocardiography (ECG) and respiratory measures to capture fast neural dynamics and autonomic regulation during three randomized auditory conditions: mindfulness (body scan), relaxation (safe place imagery), and an auditory control condition (podcast). Subjective ratings of stress and relaxation will be collected repeatedly across the procedure, complemented by questionnaires characterizing interindividual differences relevant to regulation. Outcome measures will include indices of autonomic regulation derived from cardiac activity and respiration, as well as repeated subjective ratings of stress and relaxation across conditions. Neural measures (MEG) will be used to characterize condition-related brain dynamics and brain–body coupling metrics linking neural oscillations to cardiac and respiratory rhythms. Speech-related measures during auditory guidance and brief speech-production features from post-condition reflections will be included as complementary and exploratory extensions to increase psychotherapy relevance, while accounting for methodological challenges related to overt speech in MEG. By integrating neural, physiological, and subjective measures within a single standardized paradigm, this study protocol aims to advance a mechanistic understanding of brief mind–body interventions in clinically relevant populations. Focusing on dynamic brain– body interactions during stress and regulation, the proposed approach is designed to support transparent and reproducible investigation of regulatory processes that are relevant to psychotherapy-related mind–body approaches, clinical practice, and everyday self-regulation. The findings are expected to inform future translational research and contribute to the development of mechanism-informed and potentially personalized applications of mindfulness-and relaxation-based interventions.

**Study protocol registration:**

Preregistration can be found here: https://osf.io/3rhk4/overview.

## 1 Introduction

### 1.1 Problem statement and theoretical background

Mental disorders are increasing globally and constitute one of the most pressing health challenges of modern society (WHO, 2019). Mental health, understood as the capacity to cope with stressors, realize one’s abilities, and participate productively in society, is increasingly threatened by chronic psychosocial strain (WHO, 2019). Despite the availability of effective psychotherapeutic treatments, mental health care systems remain under-resourced, and stigma continues to prevent many individuals from seeking help, sustaining persistent treatment gaps (WHO, 2019). European data underscores the magnitude of this problem. In recent surveys, nearly half of respondents reported emotional or psychosocial problems such as anxiety or depression, yet a substantial proportion did not receive professional support (European Commission, 2023). Young people appear particularly affected, reporting high levels of unmet mental health needs and marked increases in depressive symptoms during recent crises (European Commission, 2022). These developments highlight the need for interventions that are effective, scalable, and acceptable across clinical and non-clinical settings.

### 1.2 The role of mind–body interventions in mental health care

A key response to these challenges has been the growing integration of mind–body medicine (MBM) approaches that intentionally target both psychological and physiological regulation (Luberto et al., 2020; Solanki et al., 2023). MBM includes a broad set of techniques—such as mindfulness, relaxation training, guided imagery, biofeedback, yoga, and breathing-based interventions—that aim to reduce stress, improve self-regulation, and enhance quality of life with low implementation barriers and minimal side effects (Solanki et al., 2023). This mind–body perspective explicitly challenges traditional separations of “mental” versus “physical” health and emphasizes their reciprocal, dynamic interaction (Luberto et al., 2020). Such a perspective is particularly relevant given that prolonged stress without sufficient recovery contributes to dysregulation and increases risk for chronic conditions, including anxiety and depressive disorders (Schulz et al., 2023).

However, the widespread use of mind–body techniques contrast with a persistent scientific limitation: interventions are often used as interchangeable tools for “relaxation” despite conceptual differences and substantial variability in individual responsiveness (Luberto et al., 2020; Solanki et al., 2023). This is clinically consequential. If different techniques engage distinct regulatory pathways, then selecting the right tool may matter and may differ across individuals and clinical profiles.

### 1.3 From stress physiology to mechanistic targets

Stress and recovery are reflected in coordinated changes of the autonomic nervous system (ANS). The sympathetic branch supports acute mobilization, while parasympathetic activity supports recovery and restoration (Christensen et al., 2020). Beyond acute adaptation, chronic activation without adequate recovery can contribute to allostatic load, a cumulative biological burden linked to a wide range of mental and physical health outcomes (McEwen, 2002).

This study focuses on regulation as a measurable psychophysiological process and integrates brain, heart, and respiration data, with speech measures included as an optional extension. Importantly, physiological change is not always consciously perceived. Subjective experience and autonomic markers can diverge, highlighting the need to jointly assess subjective and objective indices of regulation (Mehling et al., 2018).

### 1.4 Mindfulness versus relaxation: similar outcomes, distinct regulatory strategies

Among MBM approaches, mindfulness and relaxation-based techniques are particularly prominent in clinical psychology and psychotherapy. Mindfulness is typically defined as present-moment, nonjudgmental awareness of internal experience (Kabat-Zinn, 2008) and is cultivated through exercises such as breath observation and body scan practices (Cayoun et al., 2018).

In contrast, relaxation techniques aim more directly at downregulating arousal and eliciting a measurable relaxation response (Pearson et al., 2015). A highly accessible relaxation format is guided imagery, which uses imagination and memory to evoke calming sensory experiences and promote affect regulation (Pearson et al., 2015). Clinically, safe place imagery is frequently used to support emotional safety and distress tolerance, particularly in trauma and anxiety contexts (Pearson et al., 2012, 2015).

Although both approaches are often implemented to reduce stress and arousal, they should not be mechanistically identical. Relaxation primarily targets physiological downregulation, while mindfulness emphasizes attentional and metacognitive regulation (Luberto et al., 2020). This distinction motivates a key premise of the study: similar subjective outcomes may be supported by different neurophysiological pathways, which may matter for personalization and clinical translation.

### 1.5 The missing link: brain–body interaction as a mechanistic framework

A central limitation in current evidence is that physiological indices are frequently analyzed without capturing the fast neural dynamics that initiate and shape autonomic regulation. Regulatory processes unfold over time and may rely on brain–body feedback loops rather than one-directional control. Visceral signals can shape brain dynamics and cognition, suggesting that regulation should be conceptualized as an interaction between central and peripheral processes rather than separate outcome layers (Azzalini et al., 2019).

Accordingly, this study adopts brain–body coupling as a mechanistic core. Regulation is examined not only as changes in cardiac activity or respiration and changes in neural activity, but as dynamic coordination between neural oscillations and physiological rhythms. Multimodal neuroscience methods are therefore necessary. MEG provides millisecond temporal resolution suitable for capturing rapid neural dynamics during brief interventions. The study integrates MEG with ECG and respiration to examine whether mindfulness and relaxation induce distinct neural signatures and whether these signatures couple differently to cardiac and respiratory rhythms across stress, intervention, and control contexts.

### 1.6 Why speech matters for neuroscience-based psychotherapy

Psychotherapy is fundamentally language-based. Verbal communication is not only a medium through which therapeutic interventions are delivered, but also a channel through which individuals express, organize, and reflect on internal states. In the present paradigm, spoken language is also the common delivery format of all auditory conditions. The body scan, safe place imagery, and podcast control condition are all presented as spoken audio, which means that neural responses to heard speech are an inherent part of the experimental setting. Because MEG can capture neural dynamics related to auditory speech processing with high temporal precision, speech-related neural tracking provides a relevant stimulus-engagement measure within this design. In the context of mind–body interventions, speech may provide an additional window into how participants process regulatory instructions and describe their subjective experience after regulation. At the same time, speech-related measures are methodologically challenging in MEG because overt speech can introduce movement and muscle artifacts. For this reason, speech is included in the present protocol as a complementary and carefully constrained extension of the main brain–body paradigm, rather than as the central focus of the study (Abbasi et al., 2021; Sanders, 2010). Because the podcast condition primarily requires sustained engagement with externally presented speech, whereas the body scan and safe place imagery conditions use speech to guide internally oriented regulatory processes, TRF-based neural speech tracking is expected to provide an index of stimulus engagement that may be strongest in the podcast condition.

### 1.7 Rationale and positioning of the present study protocol

Despite the widespread use of mindfulness- and relaxation-based interventions, a key limitation in the current literature is the lack of time-resolved, multimodal investigations that directly compare these approaches within the same individuals. In particular, it remains unclear how these practices shape regulation across neural dynamics, autonomic physiology, and subjective experience in individuals with mental health conditions, and how these processes are coordinated over time within a brain–body framework.

This study protocol is designed to address this gap by investigating the mechanisms of brief mind–body interventions in clinically relevant populations using a standardized, multimodal experimental paradigm. Addressing this gap is important for strengthening the mechanistic basis of psychotherapy research and for informing the future development of mechanism-informed applications of mind–body interventions.

The protocol builds on the premise that regulation is best conceptualized as a dynamic brain–body process, rather than isolated changes in subjective experience or peripheral physiology. By integrating MEG with cardiac and respiratory measures, the study aims to characterize condition-related neural signatures and their coordination with autonomic rhythms during stress, regulation, and control contexts. In addition, the study incorporates repeated subjective ratings to capture experiential regulation over time. Speech-related measures are included as an exploratory extension to enhance psychotherapy relevance while accounting for methodological constraints in MEG.

Overall, the protocol adopts a mechanistic research strategy with three complementary aims: (1) to characterize acute regulation during brief mindfulness and relaxation interventions in clinical populations, (2) to examine brain–body coupling as a dynamic marker linking neural and autonomic processes, and (3) to explore sources of interindividual variability that may inform future personalization. In doing so, the study protocol provides a transparent and reproducible foundation for subsequent translational research on mind–body interventions in mental health care.

In addition to the clinical samples targeted in this protocol phase, the preregistered NeuroPsychth program (OSF: 10.17605/OSF. IO/3RHK4) includes a healthy reference cohort assessed with the identical paradigm to provide normative benchmarks for interpreting clinical patterns; no results from any cohort are reported in this manuscript.

## 2 Aims, research questions, and hypotheses

This study aims to strengthen psychotherapy research by moving beyond outcome-focused evaluations toward a mechanistic understanding of brief mind–body interventions in clinically relevant populations, here in individuals with adult attention-deficit / hyperactivity disorder (ADHD) and depression. The long-term goal is to support the development of more differentiated, mechanism-informed, and potentially personalized applications of mindfulness- and relaxation-based interventions. The study examines how mindfulness-based and relaxation-based practices shape regulation across neural activity (MEG), autonomic physiology (cardiac activity and respiration), and subjective experience, with a particular focus on how these levels relate to each other over time. An exploratory speech component is included to increase psychotherapy relevance. Across aims, research questions, and hypotheses, analyses will first be conducted in the overall clinical sample. Where data quality and sample size permit, subgroup analyses will then be conducted within the adult ADHD and depression groups separately, and exploratory group comparisons between ADHD and depression will be examined to characterize potential clinical heterogeneity.

### 2.1 Aims

- Primary:

- To characterize acute neural and autonomic regulation during brief mindfulness-based and relaxation-based interventions.
- To differentiate mindfulness and relaxation in terms of their underlying regulatory processes.

Secondary:

- To examine brain–body coupling as a dynamic link between neural activity and autonomic rhythms.
- To examine autonomic coupling, particularly the coordination between cardiac and respiratory dynamics, across mindfulness, relaxation, and control conditions.
- To explore speech processing and brief speech production as psychotherapy-relevant extensions of the main paradigm.
- To identify psychological and lifestyle-related correlates of individual responsiveness.

### 2.2 Research questions

- Primary:

- How do mindfulness-based and relaxation-based interventions differ in their acute neural, autonomic, and experiential effects?

Secondary:

- How do neural dynamics relate over time to cardiac and respiratory regulation during stress, mindfulness, and relaxation?
- How are cardiac and respiratory dynamics coordinated across mindfulness, relaxation, and control conditions?
- When do subjective stress and relaxation ratings align with physiological and neural markers, and when do they diverge?
- Does temporal response functions (TRF)-based neural speech tracking and exploratory speech-production features differ across mindfulness, relaxation, and control conditions
- Which psychological and lifestyle characteristics are associated with stronger or weaker responsiveness to different interventions?

### 2.3 Hypotheses

#### 2.3.1 Primary

Preregistered hypotheses focus on differences between the conditions in (a) subjective stress and relaxation ratings, (b) HR, HRV (RMSSD), and respiration rate, and (c) global-band MEG power (theta, alpha, beta).

#### 2.3.2 Secondary

TRF-based neural speech tracking is included as a preregistered stimulus-engagement measure: TRF-based speech tracking is expected to be stronger in the control/podcast condition than in the mindfulness/body scan and relaxation/safe place imagery conditions. This expectation is based on the rationale that the podcast condition primarily requires sustained engagement with externally presented speech, whereas the body scan and safe place imagery conditions use spoken guidance to support internally oriented regulatory processes such as interoceptive attention, bodily awareness, or mental imagery.

Brain–body coupling outcomes are treated as preregistered non-directional mechanistic outcomes, whereas autonomic coupling extensions, time-resolved coupling extensions, speech-production features, and psychological or lifestyle correlates are treated as exploratory unless explicitly specified otherwise in the preregistration.

## 3 Methods and analysis

### 3.1 Preregistration and ethics

The present manuscript reports the clinical protocol phase of the preregistered NeuroPsychth program (OSF: 10.17605/OSF. IO/3RHK4); preregistered analyses and hypotheses are retained, with any phase-specific deviations transparently labeled. The study protocol was approved by the ethics committee of the University of Salzburg (reference number: GZ 41/2024 addendum). The study will be conducted in accordance with local legislation and institutional requirements. All participants will provide written informed consent prior to participation.

### 3.2 Study design

This study uses a prospective, within-subject (repeated-measures) experimental design with block-randomized condition order. Each participant completes one laboratory session consisting of a brief regulatory challenge (stress induction), a resting-state baseline, and three auditory conditions presented in randomized order:

- Mindfulness: body scan
- Relaxation: safe place guided imagery
- Control: podcast

**Figure 1:**
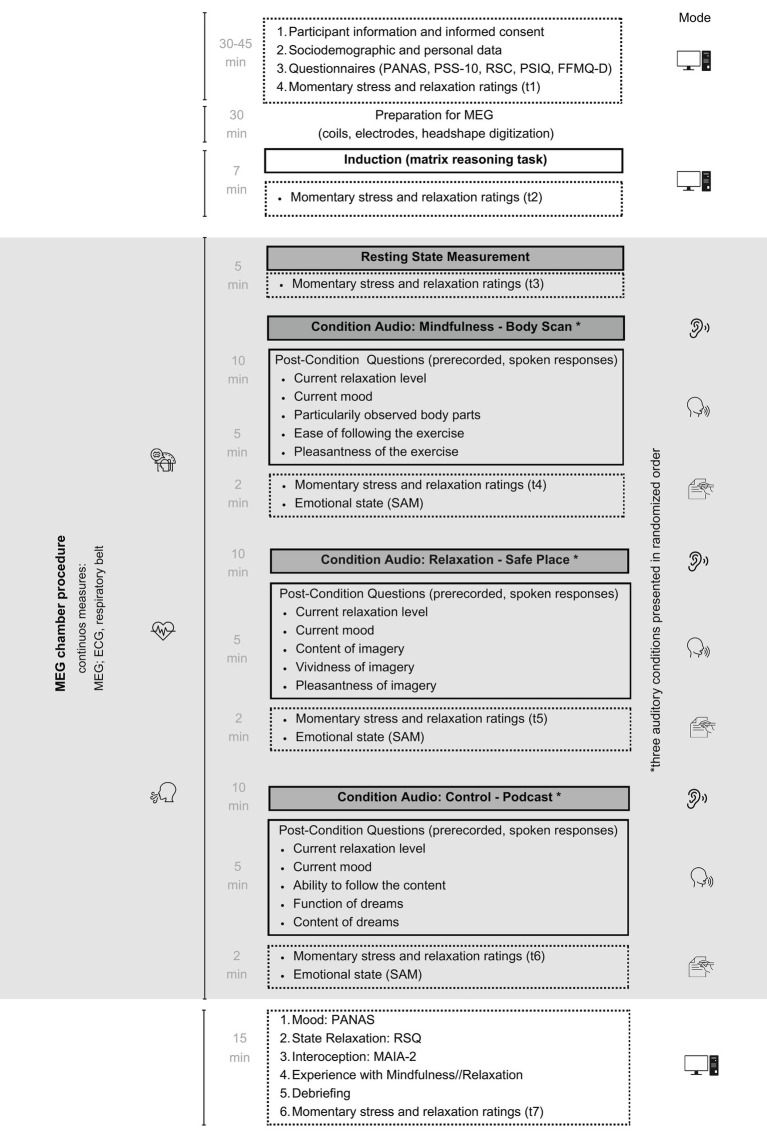
Full study procedure.

All three conditions are approximately equal in duration (∼10 min each) and are delivered as spoken audio to ensure comparable sensory input across conditions. The full study procedure is illustrated in Figure 1.

### 3.3 Randomization and blinding

The three auditory conditions are presented in randomized, counterbalanced order using a predefined condition-order scheme implemented in the MATLAB experiment script. Separate condition-order matrices are generated before data collection for the ADHD and depression groups using a random number generator. In the script, each auditory condition is assigned a numerical code: body scan = 1, safe place imagery = 2, and podcast = 3. These numbers are condition codes, not order numbers. For example, [1 2 3] means body scan–safe place imagery–podcast, whereas [2 3 1] means safe place imagery–podcast–body scan. In the ADHD matrix, for example, participant 1 could receive [1 2 3], participant 2 [1 3 2], and participant 3 [2 1 3]. At the start of the experiment, the experimenter enters the participant’s diagnostic group and sequential within-group participant number. The MATLAB script then selects the corresponding row from the predefined matrix and presents the audio files in that order. This procedure supports a balanced distribution of condition orders as far as possible within each diagnostic group and ensures that condition assignment is reproducible and documented. The selected order is saved in the participant log file.

No formal blinding is involved. Participants are not informed about the specific study hypotheses and will be debriefed after participation to minimize expectation-related effects.

### 3.4 Participants and recruitment

#### 3.4.1 Participants

Participants will be adults (≥ 18 years) from clinical populations, including individuals with depressive disorders or adult attention-deficit/hyperactivity disorder (ADHD). All participants must be fluent in German to ensure comprehension of instructions and questionnaires and must be able to provide informed consent.

#### 3.4.2 Recruitment

Participants will be recruited via social media, email, and cooperation with clinical partners and outpatient services, subject to availability and ethical approval. Interested individuals will be able to register through an online link, where they will receive initial study information and be screened for basic exclusion criteria. Individuals who appear eligible will then be invited to an appointment with trained clinical personnel at the university outpatient clinic. This appointment will be used to verify the indicated diagnosis using structured clinical diagnostic interviews, ensuring that diagnostic assessment is conducted consistently across participants.

##### 3.4.2.1 Planned sample size

Planned sample sizes are based on feasibility constraints typical for MEG studies in clinical populations and are intended to support initial mechanistic characterization rather than definitive effect size estimation. The overall clinical recruitment target is *N* = 30, with a planned minimum of *N* ≥ 15 in each diagnostic group (depressive disorders */* adult ADHD). Recruitment will continue until these targets are reached or until recruitment is no longer feasible within the project timeline.

#### 3.4.3 Diagnostic assessment

Clinical diagnoses will be established using the German clinical version of the Structured Clinical Interview for DSM-5 Disorders, Clinician Version (SCID-5-CV; Beesdo-Baum et al., 2019), conducted by trained personnel.

Current medication use is recorded for all participants, including medication type/name and dosage. This information is collected during the initial online screening and reassessed on the study day as part of the pre-experiment questionnaire to ensure accuracy and completeness. Medication use is not treated as a general exclusion criterion to reflect the clinical reality of the target populations and to support ecological validity. However, participants with recent changes in medication within the last four weeks prior to participation are excluded to reduce variability related to unstable pharmacological effects. Medication status will be considered in the statistical analyses, for example by including it as a covariate in relevant models and by conducting sensitivity analyses to assess the robustness of findings with respect to potential medication effects.

#### 3.4.4 Inclusion criteria

- Age ≥ 18 years
- Ability to provide informed consent
- Sufficient German language proficiency
- Diagnosis of depressive disorder or ADHD confirmed via SCID-5-CV interview

#### 3.4.5 Exclusion criteria

- Neurological disorders
- Contraindications for MEG recording (e.g., non-removable ferromagnetic material, implanted medical devices)
- Recent changes in medication within the last four weeks prior to participation
- Severe hearing impairment

#### 3.4.6 Healthy reference cohort (program-level)

In addition to the clinical samples described here, the preregistered NeuroPsychth program includes a healthy reference cohort assessed with the identical paradigm to provide normative benchmarks and enable integrative comparisons across health and psychopathology at the program level. No results from the healthy cohort are reported in the present manuscript.

### 3.5 Experimental procedure

Data are collected in a single laboratory session conducted in the MEG facility. After informed consent, participants complete baseline questionnaires assessing demographic, psychological, and lifestyle variables. A brief stress induction task is administered, followed by a resting-state baseline period.

The stress induction task design is informed by the Salzburg Mobile Stress Induction (SMSI), in which matrix reasoning tasks have been shown to elicit stress responses, including increases in negative affect (Vikoler et al., 2025). Prior findings indicate that stress-related changes already emerge after the first task block, supporting the use of a brief, single-block implementation in the present study. Stress is induced in the current study using matrix reasoning items. The matrix reasoning items period lasts 144 s and consists of one block with 12 items, each presented for 12 s under time pressure. In each trial, participants are required to complete an incomplete visual pattern by selecting the correct option from multiple alternatives, consistent with standard matrix reasoning paradigms. Immediate performance feedback is provided after each trial in the form of salient visual feedback indicating correct or incorrect responses, thereby increasing evaluative pressure and task engagement. The total stress induction task lasts up to approximately 5 min, including instructions, an example item, feedback, and transitions. This duration is given as an approximate upper limit because some elements are participant-paced, including reading the instructions, completing the example item, and advancing through feedback screens. In addition, the task is introduced as an intelligence-related performance task during the study briefing to further enhance its evaluative character. Full debriefing is provided at the end of the experiment.

The stress induction task is intentionally designed to be brief and moderate intensity. This reflects the aim of the study to investigate regulation processes following a controlled perturbation rather than prolonged stress exposure. A mild stressor is intended to induce a transient increase in arousal while minimizing fatigue and participant burden, which is particularly important when working with clinical populations. Furthermore, the stress induction task serves a methodological purpose by reducing interindividual variability in baseline arousal. By bringing participants to a more comparable initial physiological and affective state prior to the intervention phase, it facilitates the interpretation of condition-related changes across neural, autonomic, and subjective measures.

Because the stress induction task is administered prior to MEG recording, task-related movement or response behavior during the task does not directly contaminate the subsequent MEG recordings. To ensure participant safety and tolerability, participants are explicitly asked after the stress induction whether they feel able to continue the study.

In addition, subjective stress and relaxation levels are assessed at predefined time points to monitor the effectiveness and acceptability of the manipulation.

Participants then complete three auditory conditions—mindfulness (body scan), relaxation (safe place imagery), and a control condition (podcast)—presented in randomized order. Each condition lasts approximately 10 min. During the entire session, magnetoencephalography (MEG), cardiac activity (electrocardiography, ECG), and respiration (respiratory belt) are recorded continuously.

After each auditory condition, participants provide brief verbal responses to standardized reflection questions and complete momentary ratings of stress and relaxation at predefined time points. At the end of the session, participants complete additional questionnaires and are debriefed.

### 3.6 Standardization and auditory conditions

All auditory recordings are presented using standardized voice characteristics to minimize variability unrelated to the experimental manipulation. The same speaker’s voice and recording quality are used across all conditions. All auditory stimuli are approximately equal in duration (10 min), presented as spoken audio, and matched in general recording format. They differ primarily in their regulatory content and instructions.

The intervention duration was set to approximately 10 min for both mindfulness and relaxation exercises to align with prior work on brief mind–body interventions and to remain feasible in the MEG environment. Single short-session mindfulness interventions have been shown to affect state mindfulness, perceived stress, attention, and affect, and recent evidence suggests that 10-min and 20-min mindfulness meditations may produce comparable improvements in state mindfulness, supporting the use of a 10-min format to assess short-term state effects while minimizing participant burden (Ueberholz and Fiocco, 2022; Palmer et al., 2023; Luberto et al., 2020).

#### 3.6.1 Mindfulness condition (body scan)

The mindfulness condition consists of a guided body scan exercise based on standardized mindfulness material. Participants are guided to systematically direct attention to different body regions and to observe bodily sensations in a present-focused and non-judgmental manner. The exercise encourages participants to notice bodily sensations without attempting to change them and to gently return attention to the body when distracted. This condition is intended to target interoceptive awareness, attentional regulation, and present-centered awareness.

#### 3.6.2 Relaxation condition (safe place imagery)

The relaxation condition consists of a guided safe place imagery exercise adapted from Reddemann’s safe place procedure. Participants are instructed to imagine a personally meaningful place where they can feel safe, protected, and comfortable. Narration encourages the use of multiple sensory modalities, including visual, auditory, tactile, and emotional imagery, to increase the vividness of the imagined place. This condition is intended to support relaxation, emotional safety, and imagery-based affect regulation.

#### 3.6.3 Control condition (podcast)

The control condition consists of a spoken informational podcast presented in a calm, conversational voice. The selected segment discusses a general-interest scientific topic and is intended to provide coherent spoken input without guiding participants toward mindfulness, relaxation, imagery, breathing, or emotional regulation. The podcast therefore serves as a non-regulatory auditory control condition rather than an intervention condition. The podcast condition is matched to the mindfulness body scan and relaxation guided imagery conditions in duration, auditory modality, and general speech characteristics, including continuous spoken language, similar pacing, and speaker characteristics, while differing specifically in the absence of explicit regulatory instructions. This allows speech-related neural responses to be assessed across conditions using comparable spoken input without an explicit mindfulness, imagery, breathing, or relaxation component. The control condition therefore accounts for non-specific effects associated with listening to spoken audio, including auditory stimulation, sustained attention, and language processing, while focusing the comparison on intervention-specific regulatory processes such as attentional focus, interoceptive awareness, or imagery-based modulation.

The use of structurally matched audio conditions as controls is consistent with experimental designs in mindfulness and relaxation research, where non-regulatory spoken material, such as documentary or general-interest content, is used to control for sensory and attentional engagement without inducing targeted regulation processes (Palmer et al., 2023; Vieth and von Stockhausen, 2022; Yang et al., 2022).

### 3.7 Measures

#### 3.7.1 Data acquisition and synchronization

Neural, physiological, auditory, and behavioral data are acquired within a synchronized experimental setup. Auditory stimulus presentation, condition randomization, event timing, and trigger transmission to the magnetoencephalography (MEG) system are controlled using MATLAB, the Objective Psychophysics Toolbox, and Psychtoolbox 3 (Brainard, 1997; Pelli, 1997; Kleiner et al., 2007; Hartmann and Weisz, 2020). Brain activity is recorded in a magnetically shielded room using a 306-channel Elekta Neuromag TRIUX MEG system, comprising 102 magnetometers and 204 planar gradiometers. MEG data are sampled at 1000 Hz and filtered online between 0.1 and 330 Hz. Head position is monitored using five head-position indicator coils. Anatomical landmarks, including the nasion and left and right preauricular points, coil positions, and additional head-shape points are digitized using a Polhemus FASTRAK system. Recordings are processed with MaxFilter using signal space separation to reduce external noise and align recordings to a standard head position (Taulu et al., 2004; Taulu and Simola, 2006).

Physiological signals are recorded simultaneously using the MEG system’s built-in biochannels at 1000 Hz. Electrocardiography (ECG) is recorded using two electrodes placed on the right collarbone and the left side of the ribcage, with a ground electrode placed between the shoulder blades at neck height. Respiration is measured using a respiratory belt fastened around the abdomen. Electrooculography (EOG) is recorded using electrodes positioned above and below the right eye and at the outer canthi to support artifact identification. Auditory stimuli are presented through MEG-compatible in-ear headphones, and verbal responses are recorded using a microphone mounted inside the MEG room and documented in written form by the researcher.

#### 3.7.2 Neural measures

Neural activity is recorded continuously using MEG to capture fast neural dynamics during the resting baseline and all auditory conditions following the stress induction. Neural outcomes include spectral power estimates in predefined frequency bands relevant to regulatory processes, including alpha (8–13 Hz), theta (4–7 Hz), and beta (13–30 Hz), summarized using predefined sensor-level summaries (e.g., global and region-of-interest averages). Exploratory analyses may additionally examine condition-related differences within predefined sensor- or source-level regions of interest (e.g., frontal/midline, temporal/auditory, parietal/posterior), depending on the specific analysis plan. In addition, TRF-based neural speech-tracking analyses will relate acoustic features of the auditory stimuli to MEG activity, providing an index of neural tracking of the spoken audio material.

#### 3.7.3 Autonomic physiological measures

Cardiac activity (ECG) and respiration (respiratory belt) are recorded simultaneously with MEG to quantify autonomic regulation across the paradigm. Derived autonomic measures include heart rate variability (HRV; time-domain indices such as RMSSD), mean heart rate (HR; beats per minute), and respiration rate (RespRate; breaths per minute), calculated for each experimental phase/condition. The cardiac and respiratory time series may also support exploratory analyses of autonomic coupling, such as heart–breathing coordination across experimental conditions and provide the physiological basis for brain–body coupling analyses.

#### 3.7.4 Brain–body interaction measures (brain–body coupling)

Brain–body coupling between MEG dynamics and autonomic physiology will be assessed using complementary approaches. First, coupling will be quantified using heartbeat-locked (R-peak-locked) MEG analyses and respiration-locked MEG analyses, in which MEG activity is time-locked to cardiac R-peaks and respiratory phase or peak events to derive condition-specific coupling indices (e.g., event-locked responses and/or oscillatory power changes around cardiac and respiratory cycles). Second, as an exploratory time-resolved extension, coupling will be examined using dynamic multivariate time-series approaches that integrate neural, cardiac, and respiratory time series. These approaches will be used to characterize lagged statistical dependencies between time-resolved MEG features (e.g., band-limited power envelopes in theta/alpha/beta, extracted per sensor or ROI) and cardiac/respiratory signals, while explicitly avoiding causal interpretation. The exact modeling approach will be selected based on data quality, model assumptions, and the temporal structure of the derived neural and physiological signals. Brain–body coupling analyses will be treated as preregistered, non-directional mechanistic analyses of condition-related differences. Exploratory extensions, including time-resolved coupling approaches, will be clearly labeled as such, and multiple-comparison control will be applied for high-dimensional neural outcomes where applicable.

#### 3.7.5 Subjective measures

Momentary subjective stress and relaxation are assessed repeatedly using single-item Likert-type ratings from 0 to 10 at predefined time points, including upon arrival, after stress induction, baseline, each auditory condition, and at the end of the session. Emotional state during the MEG procedure is additionally assessed using the Self-Assessment Manikin (SAM; Bradley and Lang, 1994), a pictorial measure of affective valence, arousal, and dominance. Before the MEG recording, participants complete questionnaires assessing sociodemographic and personal data, positive and negative affect using the German version of the Positive and Negative Affect Schedule (PANAS; Breyer and Bluemke, 2016), perceived stress using the German version of the Perceived Stress Scale (PSS-10; Schneider et al., 2020), relaxation state using the German Relaxation State Questionnaire (RSQ; Steghaus and Poth, 2022), imagery ability using the Plymouth Sensory Imagery Questionnaire (PSIQ; Andrade et al., 2014), and dispositional mindfulness using the German version of the Five Facet Mindfulness Questionnaire (FFMQ-D; Michalak et al., 2016). After the MEG recording, participants again complete measures of mood and relaxation state (PANAS, RSQ), report prior experience with mindfulness and relaxation exercises, and complete the Multidimensional Assessment of Interoceptive Awareness, Version 2 (MAIA-2; Mehling et al., 2018), to assess general interoceptive awareness. These measures are used to characterize the sample and to explore interindividual variability in subjective, autonomic, neural, and brain–body regulation outcomes.

#### 3.7.6 Speech-related measures

After each auditory condition, participants provide brief spoken responses to standardized reflection questions. Speech-related analyses will be exploratory and include two components. First, neural speech tracking during auditory stimulation will be examined using temporal response functions (TRFs), which relate acoustic features of the auditory stimuli to MEG activity and provide an index of neural tracking of spoken audio material. TRF-based neural speech tracking is included as a preregistered stimulus-engagement measure. Second, speech-production features extracted from brief post-condition verbal responses, such as speech rate, pause characteristics, pitch/F0, and voice quality measures, may be analyzed depending on recording quality and the structure of the extracted speech data. These speech-production analyses are exploratory and will be interpreted cautiously, particularly because overt speech can introduce movement and muscle artifacts in MEG recordings. Therefore, speech-production analyses require rigorous artifact handling to support valid interpretation (Abbasi et al., 2021).

#### 3.7.7 Preprocessing and data quality

MEG preprocessing will follow established procedures for continuous MEG data and will be implemented using Python-based analysis tools, including MNE-Python (Gramfort et al., 2013). Preprocessing will include signal-space-separation-based noise reduction, filtering between 0.1 and 40 Hz, identification and correction of ocular and cardiac artifacts using independent component analysis, resampling to 100 Hz, and segmentation of the continuous data into condition-specific analysis windows. Components related to cardiac and ocular activity will be identified using the simultaneously recorded ECG and EOG signals and inspected as part of quality control.

For spectral analyses, power spectra will be estimated using Welch’s method, a Fourier-based approach that provides a stable estimate of how signal power is distributed across frequencies (Welch, 1967). These will be summarized within the predefined frequency bands relevant to the preregistered hypotheses. These band-limited power estimates will be averaged across sensors for global analyses and, where applicable, summarized within predefined sensor-level or source-level regions of interest. For neural speech-tracking analyses, preprocessed MEG data will be aligned with the auditory stimulus material and prepared for temporal response function analyses.

Physiological preprocessing will be conducted on the temporally aligned ECG and respiration recordings using Python-based signal-processing tools, including NeuroKit2 (Makowski et al., 2021). ECG preprocessing will include signal cleaning, optional signal inversion where required, R-peak detection, artifact correction of detected peaks, and derivation of interbeat intervals. Heart rate will be calculated from the corrected R-peak series, and HRV will be quantified using time-domain measures, with RMSSD used as the primary HRV metric. Respiration preprocessing will include respiratory signal processing, detection of respiration peaks, and calculation of respiration rate. Physiological features will be extracted for each experimental condition and, where appropriate, additionally summarized in 30-s segments to support data-quality inspection.

Data quality will be assessed separately for neural, cardiac, and respiratory outcomes. Quality-control plots of ECG, respiration, detected peaks, and segment-level physiological values will be visually inspected to identify technical failures, unreliable peak detection, or physiologically implausible values. Missing or unusable data will be identified using outcome-specific quality criteria, including technical recording failure, missing or corrupted channels, excessive MEG artifacts, unusable ECG or respiration signals, unreliable physiological feature extraction, and physiologically implausible values. Data exclusions and available sample sizes will be reported separately for each outcome. When condition-level data are incomplete but sufficient valid data remain for a participant, linear mixed-effects models may be used to retain these participants without requiring complete data for all conditions.

### 3.8 Analytical logic

The study design supports four complementary analytical levels:

- Condition-related changes: differences in neural, autonomic, and subjective measures between mindfulness, relaxation, and control conditions.
- Brain–body relationships: Mechanistic analyses of coupling between neural dynamics and cardiac and respiratory rhythms across experimental phases.
- Interindividual variability: exploratory associations between questionnaire-based psychological and lifestyle characteristics and regulation profiles.
- Speech-related analyses: associations between auditory stimulus features, neural speech tracking, and exploratory acoustic features of brief post-condition verbal responses.

### 3.9 Statistical analysis plan (overview)

Condition will be modeled as a within-subject factor with three levels: mindfulness/body scan, relaxation/safe place imagery, and control/podcast. The choice of statistical procedure will depend on the structure and distribution of the measures derived, for example multilevel models, parametric tests, or non-parametric tests. Repeated-measures ANOVA will be used for complete within-subject datasets when model assumptions are sufficiently met. Linear mixed-effects models will be used when appropriate to account for incomplete condition-level data, unequal numbers of usable observations, participant-level variability, or additional covariates such as condition order or medication status. If substantial deviations from model assumptions occur, for example due to pronounced non-normality, influential outliers, or distributional characteristics that are not adequately addressed by standard corrections or transformations, robust or non-parametric sensitivity analyses may be considered and explicitly reported.

#### 3.9.1 Exploratory and mechanistic analyses

Mechanistic and exploratory analyses will examine neural dynamics, autonomic coupling, brain–body coupling metrics, and speech-related variables. Exploratory autonomic coupling analyses may examine associations between cardiac and respiratory dynamics, such as temporal relationships between HR, HRV, and respiration, to characterize heart–breathing coordination across conditions. These analyses will be treated as exploratory and hypothesis-generating.

Brain–body coupling analyses will include heartbeat-locked (R-peak-locked) MEG analyses, respiration-locked MEG analyses, and exploratory time-resolved coupling approaches integrating MEG, cardiac, and respiratory signals. These analyses will be used to characterize statistical dependencies between time-resolved MEG features and autonomic physiology, without making causal claims.

TRF-based neural speech tracking will be analyzed as a preregistered stimulus-engagement measure. Speech-production analyses may focus on acoustic features of brief post-condition verbal responses, such as speech rate, pause characteristics, pitch/F0, and voice quality measures, depending on recording quality and the structure of the extracted speech data. Speech-production analyses will be explicitly labeled as exploratory and interpreted accordingly. These analyses will not be interpreted as primary tests of intervention efficacy.

#### 3.9.2 Missing data and data quality

Procedures for handling missing or unusable data, for example due to artifacts, will be transparently specified and reported for each outcome. Missing or unusable data will be identified using outcome-specific quality criteria, including technical recording failure, missing or corrupted channels, excessive MEG artifacts, unusable ECG or respiration signals, unreliable physiological feature extraction and physiologically implausible values. Analyses will be conducted on available data, with transparent reporting of data exclusion and sample sizes for each outcome. When condition-level data are incomplete but sufficient valid data remain for a participant, linear mixed-effects models may be used to retain these participants without requiring complete data for all conditions.

#### 3.9.3 Multiple comparisons and inference criteria

Statistical significance will be evaluated using two-tailed tests with an alpha level of *p* < 0.05. For planned pairwise condition comparisons following significant omnibus tests, *p* values will be corrected using appropriate family-wise error correction procedures, such as Bonferroni or Holm-Bonferroni correction. To account for multiple comparisons across broader sets of outcomes or features, appropriate correction procedures will be applied, particularly for neural measures involving multiple frequencies, sensors, time windows, or regions of interest, for example false discovery rate (FDR) correction or cluster-based permutation approaches where applicable. Where correction procedures are not fully specified in the preregistration, the selected correction approach will be justified and transparently reported for each analysis family.

#### 3.9.4 Program-level and clinical subgroup analyses

Program-level analyses will additionally compare clinical cohorts to a healthy reference cohort using mixed-effects models with condition as the within-subject factor and group as the between-subject factor; these analyses are not reported in the present study protocol. Within the clinical protocol phase, primary clinical analyses will first characterize condition effects across the combined clinical sample. Clinical subgroup analyses comparing depression and ADHD correspond to preregistered non-directional population-comparison analyses. Given feasibility constraints and the expected heterogeneity of clinical samples, these analyses will be interpreted cautiously and primarily as initial mechanistic subgroup characterizations rather than definitive diagnostic comparisons.

## 4 Discussion

### 4.1 Study contribution and rationale

This study protocol specifies a standardized, within-subject experimental framework to examine acute regulation after stress induction during brief mindfulness- and relaxation-based interventions in clinical populations. By combining magnetoencephalography with cardiac activity, respiration, and repeated subjective ratings within a single session, the protocol is designed to characterize condition-related regulation across neural, autonomic, and experiential levels under tightly controlled conditions. The focus is on short, commonly used interventions delivered in a standardized auditory format, allowing differentiation between mindfulness, relaxation, and control conditions within the same individuals.

### 4.2 Methodological strengths

A central strength of the protocol is the integration of multimodal measurements acquired simultaneously across conditions. This enables time-resolved analyses of neural dynamics and autonomic regulation, as well as exploratory investigation of autonomic coupling and brain–body coupling between neural oscillations and cardiac and respiratory rhythms. The within-subject design reduces interindividual confounding and supports direct condition contrasts. Standardization of auditory material, including consistent voice characteristics across conditions, is intended to minimize non-specific stimulus-related variability.

Repeated subjective stress and relaxation ratings allow experiential regulation to be assessed alongside physiological and neural measures without assuming correspondence across levels. In addition, speech-related measures provide a psychotherapy-relevant extension to the main paradigm. Neural speech tracking during auditory guidance is considered a stimulus-engagement measure, whereas speech-production features from post-condition reflections are treated as exploratory and remain methodologically constrained. Exploratory psychological and lifestyle variables are included to characterize interindividual variability and to support hypothesis generation regarding differential responsiveness. At the program level, an additional healthy reference cohort assessed with the identical paradigm will provide normative benchmarks for interpreting clinical patterns.

### 4.3 Anticipated limitations and feasibility considerations

Several limitations are inherent to the proposed design. MEG recordings impose constraints on movement and overt speech, requiring rigorous artifact handling and limiting the interpretability of speech-related analyses, which are therefore treated as exploratory. Clinical populations are heterogeneous, and recruitment feasibility may constrain sample sizes, particularly for subgroup analyses. Consequently, population-level comparisons are intended to provide initial mechanistic characterization rather than definitive diagnostic differentiation. Furthermore, the single-session design focuses on acute regulatory processes and does not address longer-term intervention effects or clinical change over time.

In addition, the protocol includes a single standardized stress induction administered prior to the intervention phase, framed as a regulatory challenge to elicit a comparable initial perturbation across participants, to avoid repeated stress exposure in clinical populations and to maintain feasibility within a single-session MEG protocol. Because the stress induction occurs once and the three auditory conditions unfold afterwards in a randomized way, time-related effects such as recovery from the initial challenge, habituation, fatigue, or carryover across the session cannot be fully excluded. The block-randomized condition order is intended to reduce systematic bias by distributing such time-related effects across mindfulness, relaxation, and control conditions; however, residual carryover effects remain a consideration when interpreting condition-related differences within the acute experimental context.

### 4.4 Scope of inference

This study protocol is not designed to directly evaluate the efficacy of psychotherapeutic interventions, treatment outcomes, or clinical effectiveness. Instead, all analyses are intended to characterize acute regulation within the experimental paradigm at neural, autonomic, and experiential levels, thereby providing a mechanistic description of how brief, commonly used regulatory strategies engage brain and body systems in clinical populations. Exploratory components, including autonomic as well as brain–body coupling, speech-production features, and psychological and lifestyle correlates, will be explicitly labeled as such. Brain–body coupling outcomes and TRF-based neural speech tracking will be reported according to their preregistered status. Findings will be interpreted mechanistically and descriptively, without direct causal or therapeutic claims, and will be used to generate testable hypotheses about differential responsiveness, including “what works for whom and why,” to be evaluated in future outcome-focused designs.

### 4.5 Implications for future research

By providing a preregistered and transparent multimodal framework, this protocol is intended to support subsequent hypothesis-driven and intervention-focused studies that bridge mechanistic markers to psychotherapy-relevant endpoints. The design may serve as a foundation for future work, extending the acute paradigm to other psychotherapeutic intervention contexts, additional clinical populations, and personalization-oriented questions. Specifically, identifying condition-specific neural–autonomic signatures and brain–body coupling profiles may enable mechanistically informed hypotheses about individual differences in responsiveness to brief regulatory strategies.

## 5 Ethics and dissemination

The study protocol has been approved by the ethics committee of the University of Salzburg (reference number: GZ41/2024 addendum). The study will be conducted in accordance with applicable local and institutional regulations. An emergency and crisis management plan is in place for acute psychological or physical distress during or after participation. Participants may pause or terminate participation at any time without negative consequences. In case of marked distress, MEG recording will be stopped immediately, and the participant will be supported by trained study personnel; if crisis indicators persist, participation will be terminated and on-site clinical staff at the Clinic will be contacted for further assessment and care. Contact information for crisis services is available in the laboratory and provided to participants as part of the study information material.

The study protocol has been preregistered on the Open Science Framework (OSF; Registration DOI: 10.17605/OSF. IO/3RHK4) to promote transparency and reproducibility. Results will be disseminated through peer-reviewed scientific publications and presentations at national and international conferences. Findings will be reported at the group level only, with a clear distinction between primary and exploratory analyses. Any deviations from the preregistered analysis plan will be transparently documented and justified in subsequent publications. Where appropriate and ethically permissible, anonymized data and analysis code may be shared in accordance with open science practices, taking particular care with respect to clinical and speech-related data. Dissemination will focus on mechanistic insights into regulation and brain–body interaction during brief mind–body interventions, providing a foundation for future hypothesis-driven and translational research rather than direct clinical recommendations.
